# Symphysis-Fundal Height Curve in Pregnancies Complicated by Maternal Hyperglycemia: Comparison with Curves of Nondiabetic Pregnant Women

**DOI:** 10.1155/2020/1908764

**Published:** 2020-09-01

**Authors:** Neusa A. S. Basso, Roberto A. A. Costa, Adriano Dias, Claudia G. Magalhães, Marilza V. C. Rudge, Iracema M. P. Calderon

**Affiliations:** ^1^Graduate Program in Gynecology, Obstetrics and Mastology/Botucatu Medical School/UNESP, SP, Brazil; ^2^Department of Gynecology and Obstetrics, Botucatu Medical School/São Paulo State University/UNESP-Botucatu, SP, Brazil; ^3^Department of Public Health, Botucatu Medical School/São Paulo State University/UNESP-Botucatu, SP, Brazil

## Abstract

**Background:**

Reference symphysis-fundal height (SFH) curves for pregnancies complicated by maternal hyperglycemia are not available.

**Objective:**

To build an SFH curve according to gestational age for pregnant women with hyperglycemia-type 2 diabetes (T2DM), gestational diabetes mellitus (GDM), or mild gestational hyperglycemia (MGH) and compare it with three other curves in use in Brazil.

**Methods:**

Prospective cohort study of 422 pregnant women with hyperglycemia attending the Perinatal Diabetes Research Center (PDRC) of Botucatu Medical School, São Paulo State University/UNESP. Between 13 and 41 weeks of pregnancy, 2470 SFH measurements were obtained (mean 5.85 per woman). For the assessment of glycemic control, 2074 glucose level measurements were taken and the glycemic mean (GM) at each gestational week was estimated.

**Results:**

GM was adequate (<120 mg/dL) in 94.9% and inadequate (≥120 mg/dL) in 5.1% of the cases. The equation applied for SFH prediction was expressed as SFH = 1.082 + 0.966∗week (*r*^2^ = 84.6%). At visual analysis, P10 and P90 SFH measurements were higher in the study curve than in the three other curves. Statistical analysis confirmed that SFH median values in this study were higher than those in the reference curve of habitual risk pregnancies, especially after 19 weeks of pregnancy.

**Conclusion:**

Taking into account that the maternal hyperglycemia was at strict control, our unedited results suggest that the current SFH curve can be a useful tool in prenatal care of T2DM, GDM, and MGH pregnant women.

## 1. Introduction

Symphysis-fundal height (SFH) measurement is a simple method to assess fetal growth in relation to gestational age (GA) that can detect twin pregnancies, polyhydramnios, oligohydramnios, and other complications. Ultrasound may be an accurate tool to detect fetal growth restriction (FGR) and macrosomia, with sensitivity at 93% and 90%, respectively. However, the SFH measurement is a current universal practice, and a change from what is usually practiced in a particular setting is not recommended [1, 2].

In the Brazilian Public Health System (SUS), the reference SFH chart was developed by Fescina et al., based on measurements taken from 47 Paraguayan pregnant women [3, 4].

Owing to population differences, some authors advocate that charts should be locally generated for best results, and specific SFH curves were produced from Brazilian pregnant women. Oppermann et al. [5] constructed a curve based on measurements from 3539 low-risk pregnant Brazilian women and compared it with the curve built by Fescina et al. [4]. They concluded that the chart of Fescina et al. [4] does not reflect the current pattern of uterine growth in pregnant Brazilian women and is, therefore, not appropriate to detect abnormal fetal growth, especially intrauterine growth restriction [5]. Likewise, Freire et al. [6] built a curve of fundal height according to gestational age among 227 low-risk pregnant women and also compared it with the chart of Fescina et al. [4]. The authors observed that mean uterine height significantly differed from 19 weeks of pregnancy onward, suggesting differences between curves when used for screening fetal growth deviations [6].

Notwithstanding their differences in performance, these current standards are unlikely to be a suitable reference for both low-risk and high-risk populations. Gestational diabetes mellitus (GDM) and preeclampsia are common complications of pregnancy that are known to be associated with adverse perinatal outcomes such as macrosomia, fetal growth restriction (FGR), low birthweight, and consequent higher risk of perinatal death [7–11]. However, reference SFH curves for pregnancies complicated by maternal hyperglycemia are not available. This study was aimed at building an SFH curve according to gestational age among pregnant women with hyperglycemia and comparing it with the Brazilian reference curves.

## 2. Methods

This study was carried out at the Perinatal Diabetes Research Center (PDRC) of Botucatu Medical School, São Paulo State University/UNESP, and approved by Institutional Review Board of the Botucatu Medical School/UNESP (CEP-FMB/UNESP #255/08). Assuming a prevalence of gestational hyperglycemic disorders of 12%, the minimum sample size was estimated at 165 subjects and at least 13 weekly SFH measurements during pregnancy.

**Figure 1:**
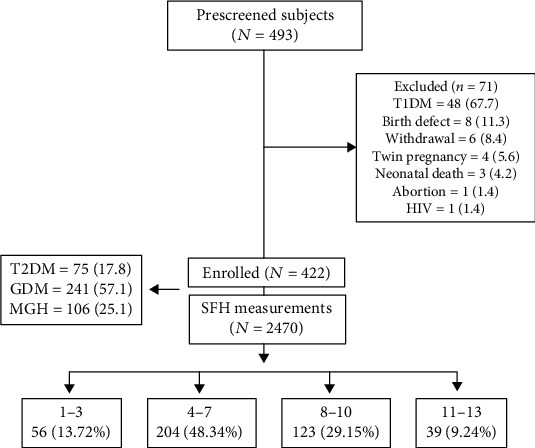
Flow chart of study participants according to inclusion and exclusion criteria.

In the current study, 422 pregnant women were enrolled and 2470 SFH measurements were obtained. Inclusion criteria were as follows: type 2 diabetes mellitus (T2DM) without micro- or macrovascular diseases (classes B and C of Priscilla White), gestational diabetes mellitus (GDM), or mild gestational hyperglycemia (MGH); receive prenatal and labor care at our center; gestational age confirmed by ultrasound before 20 weeks of pregnancy; live singleton pregnancy; and written informed consent. Exclusion criteria were as follows: birth defects detected during pregnancy; type 1 diabetes mellitus (T1DM); and associated consumptive disease. The study flowchart is in Figure 1.

Using a pretested protocol designed for the study, data were collected via review of medical records and semistructured interviews and included information on age (years), race (white/nonwhite), body mass index (BMI; kg/m^2^), smoking status (yes/no), number of previous pregnancies, C-sections and abortions, gestational age at birth, and glycemic mean (GM).

SFH was measured according to the standard technique recommended by the Brazilian Ministry of Health [3]. Immediately after emptying the bladder, the zero mark of a flexible inelastic tape measure was placed at the uppermost border of the pubic symphysis, and the tape was extended to the uterine fundus. The distance from the top of the symphysis pubis to the depression in front of the pad of the middle finger was measured, recorded, and related with gestational age confirmed by ultrasound. The arithmetic mean of three consecutive measurements was used during linear regression analysis.

All subjects with hyperglycemia included in this cohort started prenatal care before 20 weeks of gestation and, except for T2DM, which was previously identified, underwent maternal hyperglycemia screening protocol between 24 and 28 gestational weeks. GDM diagnostic test was 75 g OGTT-FPG between 5.1 and 6.9 mmol/L (92–125 mg/dL) or 1 h postload plasma glucose equal or above 10.0 mmol/L (180 mg/dL) or 2 h postload plasma glucose between 8.5 and 11.0 mmol/L (153–199 mg/dL) [12, 13]; the criteria for MGH diagnosis were normal 75 g OGTT and altered GP test, that is, fasting plasma glucose equal or above 90 mg/dL (5.0 mmol/L) or 2 h postprandial plasma glucose equal or above 130 mg/dL (7.2 mmol/L). The GP test was performed over a one-day hospital stay with the women on a 2840 kcal diet, fractionated in five meals. Plasma glucose measurement was taken every two hours, from 8 AM to 6 PM [14]. Immediately after the diagnosis, both MGH and GDM, as well as T2DM pregnant women previously identified, were cared for by a multiprofessional team and underwent maternal glucose control, according to the ADA's recommendation. Lifestyle changes (diet and exercise) were first recommended, and it was complemented by insulin therapy when glycemic goals were not achieved [15]. Oral hypoglycemic drugs are not recommended by the Brazilian Health Surveillance Agency (ANVISA) to be used in pregnancy, so they are not prescribed in our center. The maternal glucose control was monitored every 1 or 2 weeks by GP test, performed with an individual-specific diet [14, 15].

According to BMI, using self-reported prepregnancy weight/height^2^, subjects were classified as underweight (<18.5 kg/m^2^), adequate weight (18.6–24.9 kg/m^2^), overweight (25–29.9 kg/m^2^), obese class I (30–34.9 kg/m^2^), obese class II (35–39.9 kg/m^2^), or obese class III (≥40 kg/m^2^) [16].

GM, a marker of maternal glycemic control, was defined as the arithmetic mean of glucose level measurements taken on the day before SFH assessment in six samples obtained from subjects treated with insulin and in 10 samples from subjects not receiving insulin. Glycemic control was classified as adequate (GM < 120 mg/dL) or inadequate (GM ≥ 120 mg/dL) [14].

Statistical analyses were performed using SPSS Statistics®, v.20.0. Medians were compared using the Mann-Whitney nonparametric test as data were not normally distributed. Measures of central tendency and dispersion for fundal height were estimated for each gestational week within the interval between 13 and 41 weeks of pregnancy. Simple linear regression analysis was used to develop the predictive equation of SFH in the function of gestational age. SFH curves were compared considering gestational weeks as comparable, percentiles as known, and standard deviations as unknown but assumed as equal. The significance level was set at 95% (*p* < 0.05) for all tests.

## 3. Results

Table 1 shows the clinical and obstetric characteristics of the study population. Mean age was 30.7 years. Mean BMI was 31.0 kg/m^2^ with a range of 18.1 to 55.7 kg/m^2^. Of the 422 study participants, 62.3% were classified as either overweight or obese (BMI ≥ 25 kg/m^2^), and 14.5% were smokers. Delivery occurred at term in 80.6% of the cases.

Glycemic control was assessed through 2074 glucose level measurements and GM at each gestational week. GM was adequate (<120 mg/dL) in 94.9% and inadequate (≥120 mg/dL) in only 5.1% of the cases. Adequate glycemic control prevailed except at week 23, when GM was 123.9 mg/dL (Table 1A, supplementary material).

SFH measurements are shown in Table 2. Simple linear regression analysis demonstrated a statistically significant relationship between variables (*r*^2^ = 84.6%, *p* < 0.001) and resulted in the following equation for SFH prediction:

(1) $$\begin{array}{c} \text{SFH}=1.082+0.966*\text{week}. \end{array}$$

Table 2A (supplementary material) shows the upper and lower limits of the 95% confidence intervals (CI).

**Figure 2:**
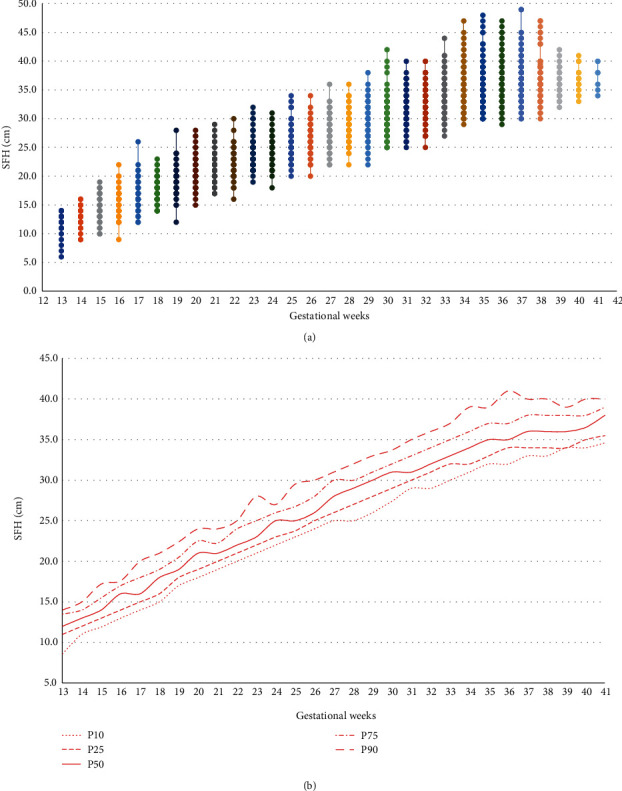
SFH curve between 13 and 41 weeks, adjusted by simple linear regression (a), and SFH percentile curve (b) among pregnant women with T2DM, GDM, and MGH.

Figure 2 shows the SFH curve adjusted by simple regression analysis (Figure 2(a)) and against percentile limits (Figure 2(b)) between 13 and 41 weeks.

**Figure 3:**
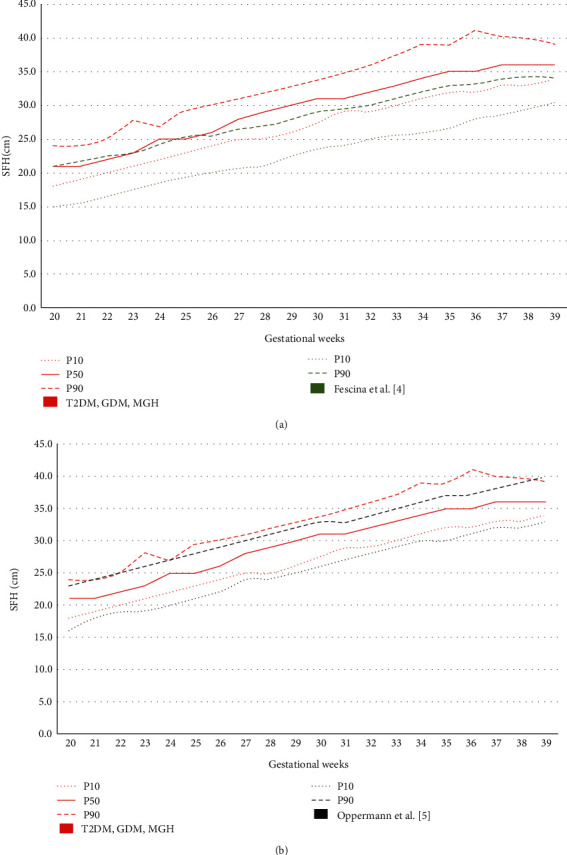
Comparison of the SFH curve among pregnant women with T2DM, GDM, and MGH with those of Fescina et al. [4] (a) and Oppermann et al. [5] (b).

**Figure 4:**
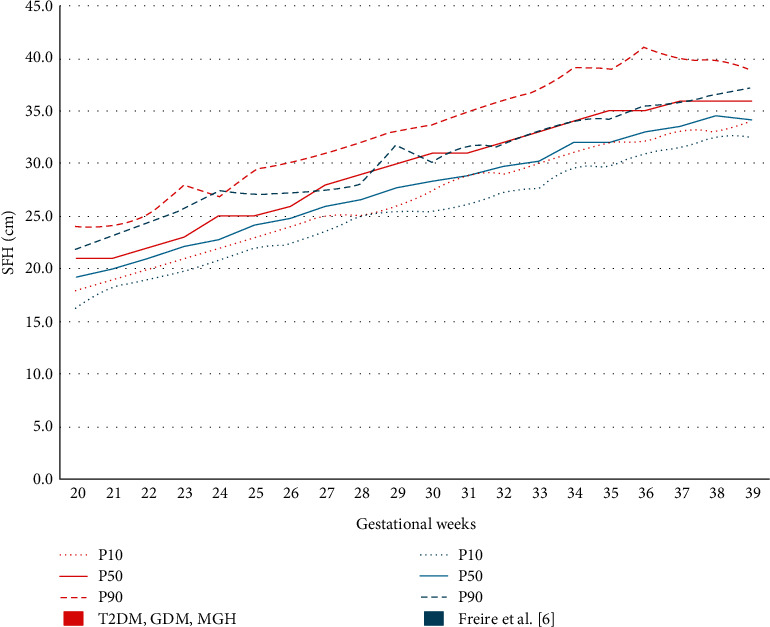
Comparison between the SFH curve among pregnant women with T2DM, GDM, and MGH and that of Freire et al. [6].

Visual comparison makes evident that P10 and P90 SFH measurements were higher in the study curve than in the curves of Fescina et al. [4] and Oppermann et al. [5] (Table 3 and Figures 3(a) and 3(b)). The same was observed when the study curve was compared with that of Freire et al. [6] (Table 4 and Figure 4).

Comparison between the study curve and the curve of Freire et al. [6] showed that P50 limits in the study curve corresponded with P90 limits in the curve of Freire et al. [6] from week 26 onward. From week 19 onward, the mean values observed in this study were higher than those found by Freire et al. [6] (Table 4 and Figure 4).

## 4. Discussion

This study included 422 pregnant women with T2DM, GDM, or MGH, who were treated according to our center's protocol. As a result, maternal glycemic mean (GM) was found to be adequate (<120 mg/dL) among them except at 13, 14, and 23 weeks of pregnancy. Thus, the study curve was built based on SFH measurements taken from pregnant women with adequately treated and controlled hyperglycemia. Tight maternal hyperglycemia control (GM < 120 mg/dL) is known to help prevent a cascade of fetal and neonatal adverse events and might control intrauterine overgrowth [14]. The consistency of the SFH measurements and results obtained here were confirmed by an adjusted linear model where *r*^2^ = 85.2%.

In comparison with the curve of Fescina et al. [4], recommended by the Brazilian Ministry of Health and currently used in our center, the curve developed in this study showed higher P10 and P90 absolute values. The same occurred when the study curve was compared with the curve of Oppermann et al. [5], which was constructed among Brazilian pregnant women at risk of developing GDM. However, comparison between these curves was qualitative and visual as a statistical analysis could not be performed because the number of measurements at each gestational week was unknown.

Comparison between the study curve and the curve of Freire et al. [6] confirmed the statistical difference. In this study, mean SFH measurements were higher from 19 to 39 weeks of pregnancy. A visual analysis revealed that P10, P50, and P90 limits were always higher than those in the curve of Freire et al. [6]. These findings raise the question of whether the curves proposed in the literature, even those developed among Brazilian pregnant women, should be used for monitoring pregnancies complicated by maternal hyperglycemia yet adequately controlled.

Differences between curves developed for Brazilian women and the standard SFH curve developed by Fescina et al. [4], which is recommended by the Brazilian Ministry of Health, have already been pointed out. These differences have been frequently attributed to methodological reasons [5, 6]. Measurement techniques, number of examiners, prior bladder emptying, and gestational age estimates have all been considered as influencing factors. In this study, similarly to that of Freire et al. [6], measurements were taken by a single observer using a standard technique, each SFH value corresponded to the arithmetic mean of three consecutive measurements, bladder emptying was observed before assessment, and gestational age was confirmed by ultrasound. However, in the study of Fescina et al. [4], SFH was measured by several observers, not all women had an empty bladder during assessment, and gestational age was calculated based on the last menstruation date. The same may be said about the study of Oppermann et al. [5] that included a multicentric population whose SFH measurements and prenatal data were extracted from hospital records.

It is worthy of note that despite adequate glycemic control, as confirmed the by the GM values lower than 120 mg/dL observed over 24 of the 27 gestational weeks, the pregnant women participating in this study had T2DM, GDM, or MGH and were mostly white (70.9%) and aged ≥25 years (81.7%) with BMI ≥ 25 kg/m^2^ (82.3%). Overweight and obesity were observed in 25.6% and 60.4% of the subjects, respectively. These characteristics alone differentiate this study from others and can explain the different SFH measurements found. Nonetheless, these same characteristics did not seem to be so relevant within the study itself; the linear regression equation for SFH prediction [SFH = 1.082 + 0.966∗gestational week] demonstrated SFH variation only in function of gestational age.

The fact that other studies including populations with characteristics similar to those seen in our subjects are not found in the literature hampers an in-depth analysis of our results. On the other hand, these same characteristics suggest the inadequacy of the currently available SFH curves to the population under study.

High sensitivity is a prerequisite for a good screening test. Nonetheless, the sensitivity of the curve of Fescina et al. [4] to detect fetal growth restriction (FGR) was 0.8-6% while that of Oppermann et al. [5] was 8-29%. In contrast, the curve of Fescina et al. [4] showed high sensitivity in detecting fetal macrosomia (70-89%), whereas the curve of Oppermann et al. [5] was less sensitive (11-21%). In a later validation study, Freire et al. [17] compared SFH measurements with a birthweight curve in a subsample of 122 pregnant women and neonates. The sensitivity of Freire's curve was higher than that of Fescina et al. [4] in diagnosing small for gestational age fetuses and lower in detecting macrosomia [17]. This once more demonstrates that, to date, no curve has been able to identify risk for both fetal overgrowth and growth restriction at the same time.

Given our greater interest in detecting fetal macrosomia, which is more common in pregnancies complicated by maternal hyperglycemia, we could keep using the curve of Fescina et al. [4] or even the Oppermann et al. curve [5] in routine practice. However, the higher percentile limits observed in the curve created in this study, as well as the adequacy of maternal glycemic control, suggest that the study curve would be a better tool for the monitoring of this specific population of pregnant women. Moreover, the current SFH curve was validated in a similar population with 206 T2DM, GDM, and MGH pregnant women and showed high performance in predicting both small (SGA) and large for gestational age (LGA) newborns, with better performance than the national reference SFH chart [18, 19].

### 4.1. Strengths and Limitations

This study has limitations. The main one is the lack of subsequent FS assessments of the same pregnant woman every week of pregnancy. Due to the prenatal logistics, we chose serial measures in an expanded population but evaluated by a single person, adequately trained and qualified for this. We try to guarantee the greatest number of measures for each pregnant woman, and we reach an average of six evaluations/pregnant woman. In contrast, the gestational age confirmed by ultrasound before 20 weeks [20], a single person performing deevaluation, and the successful results of validating the current SFH curve [18, 19] represent the strength of the study.

### 4.2. Clinical Implications

Fetal growth in high-risk pregnancies should be monitored with serial ultrasound scans by plotting anthropometric measures against international standards [20]. Unfortunately, this is not the reality in low- and middle-income countries, where the simple and inexpensive SFH measurement is a unique tool for fetal growth screening, even in high-risk pregnancies [1]. This is a common scenario in several regions in Brazil, and so the proposed SFH curve will contribute to the screening of the mother and fetus that need ultrasonography evaluation and improvement of the glucose control.

In our study, with regard to the visual comparison of the current SFH curve with the reference ones, it was observed that (i) concerning the Fescina et al. SFH chart [4], the limits of P50 and P90 are above the reference of P90; (ii) to the Freire et al. curve [6], the limits of P50 overlap the reference limits of P90 and the limits of P90 are well above the reference limits of P90; and (iii) the extreme limits (P10 and P90) of the Oppermann et al. curve [5] would be the closest and comparable to those established in the current SFH curve.

In clinical practice, the Fescina et al. SFH chart [4] is the Brazilian reference and therefore in pregnancies complicated by T2DM, GDM, or MGH could imply a large number of false-positive evaluations that would require a complementary ultrasound with a higher cost for the Brazilian Health System. Finally, the recommendation of the current SFH curve in the clinical practice is supported by its best performance to identify both SGA or LGA fetuses compared with reference curves previously published [18, 19] and by the potential economic benefit and improvement of prenatal care in these high-risk pregnancies.

## 5. Conclusion

The unedited results of this study, with strict maternal glucose control, as well as the predictive performance of the current SFH curve, suggest that it may be a useful tool in the prenatal care of T2DM, GDM, and MGH pregnant women.

## Figures and Tables

**Table 1:** Characteristics of the study population—422 pregnant women with T2DM, GDM, and MGH.

Characteristic	T2DM	GDM	MGH	Total
	*N*	*N*	*N*	*N* (%)
Age (years)				
14–19	0	3	7	10 (2.4)
20–24	7	32	28	67 (15.9)
25–29	12	52	26	90 (21.3)
30–34	30	86	27	143 (33.9)
≥35	26	68	18	112 (26.5)
Race				
White	49	167	83	299 (70.9)
Nonwhite	26	74	23	123 (29.1)
BMI				
<18.5	0	1	0	1 (0.2)
18.6–24.9	14	30	30	74 (17.5)
25–29.9	16	62	30	108 (25.6)
30–34.9	21	79	28	128 (30.4)
35–35.9	14	42	11	67 (15.9)
≥40	10	27	7	44 (10.4)
Smoking				
Yes	12	35	14	61 (14.5)
No	63	206	92	361 (85.5)
Number of pregnancies				
1	4	40	24	68 (16.1)
2	16	70	25	111 (26.3)
3	23	62	20	105 (24.9)
≥4	32	69	37	138 (32.7)
Previous C-section				
Yes	43	117	47	207 (49.1)
No	32	124	59	215 (50.9)
Abortion				
Yes	27	62	25	114 (27.0)
No	48	179	81	308 (73.0)
g.a. at delivery (weeks)				
27–31	2	2	0	4 (0.9)
32–36	23	46	9	78 (18.5)
≥37	50	193	97	340 (80.6)
Total	75	241	106	422

BMI: body mass index (WHO, 2004).

**Table 2:** SFH measurements taken between 13 and 39 of gestation from pregnant women with T2DM, GDM, and MGH.

Gestational week	Measurements (*N*)	Mean (cm)	Sd	Minimum (cm)	Maximum (cm)	Percentiles		
						P25	P50	P75
13	27	11.85	2.214	6	14	11.00	12.00	13.50
14	34	12.94	1.687	9	16	12.00	13.00	14.00
15	40	14.38	2.215	10	19	13.00	14.00	16.00
16	65	15.48	2.195	9	22	14.00	16.00	17.00
17	66	16.74	2.598	12	26	15.00	16.00	18.25
18	61	17.67	2.095	14	23	16.00	18.00	19.00
19	68	19.34	2.519	12	28	18.00	19.00	20.75
20	67	20.79	2.705	15	28	19.00	21.00	23.00
21	78	21.47	2.312	17	29	20.00	21.50	22.25
22	80	22.25	2.368	16	30	21.00	22.50	24.00
23	80	23.93	2.642	19	32	22.00	23.00	25.00
24	83	24.52	2.334	18	31	23.00	25.00	26.00
25	87	25.33	2.613	20	33	23.00	25.00	26.00
26	88	26.57	2.382	20	34	25.00	26.00	28.00
27	104	27.95	2.506	22	36	26.00	28.00	30.00
28	114	28.64	2.570	22	36	27.00	29.00	30.00
29	125	29.54	2.693	22	38	28.00	30.00	31.00
30	135	30.78	2.812	25	42	29.00	31.00	32.00
31	145	31.68	2.519	25	40	30.00	31.00	33.00
32	151	32.29	2.655	25	40	31.00	32.00	34.00
33	169	33.49	2.767	27	44	32.00	33.00	35.00
34	184	34.52	3.174	29	47	32.00	34.00	36.00
35	193	35.10	3.223	29	47	34.00	35.00	37.00
36	209	35.91	3.203	29	47	34.00	35.00	37.00
37	192	36.32	3.083	30	49	34.00	36.00	38.00
38	127	36.33	3.186	30	47	34.00	36.00	38.00
39	59	36.24	2.299	32	42	34.00	36.00	38.00

**Table 3:** Percentile 10 and 90 SFH values between 13 and 41 weeks among pregnant women with T2DM, GDM, and MGH and in Fescina et al. [4] and Oppermann et al. [5].

	T2DM, GDM, MGH		Fescina et al. [4]		Oppermann et al. [5]	
GA	P10	P90	P10	P90	P10	P90
20	18.0	24.0	15.0	21.0	16.0	23.0
21	19.0	24.0	15.5	21.5	18.0	24.0
22	20.0	25.0	16.5	22.5	19.0	25.0
23	21.0	28.0	17.5	23.0	19.0	26.0
24	22.0	27.0	18.5	24.0	20.0	27.0
25	23.0	29.5	19.5	25.5	21.0	28.0
26	24.0	30.0	20.0	25.5	22.0	29.0
27	25.0	31.0	20.5	26.5	24.0	30.0
28	25.0	32.0	21.0	27.0	24.0	31.0
29	26.0	33.0	22.5	28.0	25.0	32.0
30	27.4	33.7	23.5	29.0	26.0	33.0
31	29.0	35.0	24.0	29.5	27.0	32.9
32	29.0	36.0	25.0	30.0	28.0	34.0
33	30.0	37.0	25.5	31.0	29.0	35.0
34	31.0	39.0	26.0	32.0	30.0	36.0
35	32.0	39.0	26.5	33.0	30.0	37.0
36	32.0	41.0	28.0	33.0	31.0	37.0
37	33.0	40.0	28.5	34.0	32.0	38.0
38	33.0	40.0	29.5	34.0	32.0	39.0
39	34.0	39.0	30.5	34.0	33.0	40.0

GA: gestational age.

**Table 4:** Number of SFH measurements, SFH means, and standard deviations (sd) between 13 and 39 weeks among pregnant women with T2DM, GDM, and MGH and in Freire et al. [6].

GA	T2DM, GDM, MGH			Freire et al. [6]			*p* ^∗^
	*N*	Mean (cm)	sd	*n*	Mean (cm)	sd	
13	27	12.0	2.5	35	11.0	3.0	**0.023**
14	34	13.0	2.0	38	12.0	1.9	0.068
15	40	14.0	3.0	30	13.3	2.3	0.061
16	65	16.0	3.0	33	15.2	3.3	0.414
17	66	16.0	3.3	38	15.9	2.5	0.211
18	61	18.0	3.0	49	17.2	2.4	0.216
19	68	19.0	2.8	44	18.4	2.7	**0.014**
20	67	21.0	4.0	62	19.2	3.0	**≤0.001**
21	78	21.5	2.3	47	20.0	2.0	**≤0.001**
22	80	22.5	3.0	37	21.0	3.4	**0.039**
23	80	23.0	3.0	32	22.2	2.9	**0.008**
24	83	25.0	3.0	37	22.8	2.7	**0.015**
25	87	25.0	3.0	39	24.2	3.5	0.205
26	88	26.0	3.0	35	24.8	3.5	**≤0.001**
27	104	28.0	4.0	37	26.0	2.0	**≤0.001**
28	114	29.0	3.0	39	26.6	2.0	**≤0.001**
29	125	30.0	3.0	32	27.6	2.7	**0.003**
30	135	31.0	3.0	37	28.3	3.2	**≤0.001**
31	145	31.0	3.0	33	28.8	2.9	**≤0.001**
32	151	32.0	3.0	39	29.8	2.4	**≤0.001**
33	169	33.0	3.0	51	30.3	2.5	**≤0.001**
34	184	34.0	4.0	45	32.0	2.4	**≤0.001**
35	193	35.0	3.0	48	32.0	2.1	**≤0.001**
36	209	35.0	3.0	60	33.0	2.5	**≤0.001**
37	192	36.0	4.0	80	33.5	2.1	**≤0.001**
38	127	36.0	4.0	101	34.5	2.3	**≤0.001**
39	59	36.0	4.0	48	34.2	3.4	**≤0.001**

*n*: number of FH measurements at each week of gestation; ^∗^Student's *t*-test. The *p* values in bold have statistical significance (*p* < 0.05).

## Data Availability

Data and any supporting material regarding this manuscript are available, and they can be requested from the correspondence author at any time.

## Supplementary Materials

Supplementary Materials Table 1A: glycemic control assessed between 13 and 39 weeks among pregnant women with T2DM, GDM, and MGH. Table 2A: SFH expected value with upper and lower limits (95% CI) between 13 and 42 weeks or gestation among pregnant women with T2DM, GDM, and MGH.
