# Supplementary material for: Symphysis-Fundal Height Curve in Pregnancies Complicated by Maternal Hyperglycemia: Comparison with Curves of Nondiabetic Pregnant Women
Source: Biomed Res Int. 2020 Sep 1;2020:1908764. doi: 10.1155/2020/1908764 (PMC7481913; doi:10.1155/2020/1908764)
Supplement: Supplementary Materials — Table 1A: glycemic control assessed between 13 and 39 weeks among pregnant women with T2DM, GDM, and MGH. Table 2A: SFH expected value with upper and lower limits (95% CI) between 13 and 42 weeks or gestation among pregnant women with T2DM, GDM, and MGH. [file 1908764.f1.zip › BioMedIntRes_Supplem mat_v. final_julho2020_BMRI_3121474.docx]

**Biomed Research International**

**Symphysis-fundal height curve in pregnancies complicated by maternal hyperglycemia – comparison with curves of non-diabetic pregnant women (#1908764)**

**Supplementary materials**

**Table 1A.** Glycemic control assessed between 13 and 39 weeks among pregnant women with T2DM, GDM and MGH

| Weeks | # glucose tests | minimum | maximum | mean (mg/dL) |
| --- | --- | --- | --- | --- |
| 13 | 27 | 87.60 | 233.30 | 123.12 |
| 14 | 33 | 72.40 | 213.00 | 120.65 |
| 15 | 25 | 70.40 | 189.60 | **118.60** |
| 16 | 35 | 87.90 | 186.00 | **117.11** |
| 17 | 31 | 69.60 | 249.10 | **110.71** |
| 18 | 33 | 67.30 | 170.30 | **109.62** |
| 19 | 37 | 66.30 | 161.90 | **112.84** |
| 20 | 40 | 69.70 | 216.80 | **116.52G** |
| 21 | 42 | 73.20 | 240.00 | **111.31** |
| 22 | 46 | 80.00 | 170.80 | **115.08** |
| 23 | 46 | 72.20 | 273.50 | 123.94 |
| 24 | 69 | 76.20 | 174.00 | **111.01** |
| 25 | 76 | 76.30 | 180.80 | **112.06** |
| 26 | 88 | 79.70 | 188.50 | **110.37** |
| 27 | 83 | 75.10 | 334.70 | **113.62** |
| 28 | 116 | 56.40 | 235.50 | **111.93** |
| 29 | 89 | 79.20 | 157.30 | **108.89** |
| 30 | 110 | 54.00 | 281.80 | **109.35** |
| 31 | 109 | 72.00 | 198.00 | **109.56** |
| 32 | 139 | 61.80 | 198.20 | **109.01** |
| 33 | 145 | 76.80 | 236.30 | **106.53** |
| 34 | 149 | 52.80 | 152.50 | **104.14** |
| 35 | 167 | 67.40 | 180.90 | **100.91** |
| 36 | 163 | 58.80 | 187.50 | **99.31** |
| 37 | 124 | 64.10 | 138.00 | **98.38** |
| 38 | 41 | 77.50 | 124.00 | **100.16** |
| 39 | 11 | 81.10 | 106.30 | **94.46** |

**Table 2A.** SFH expected value with upper and lower limits (95% CI) between 13 and 42 weeks or gestation among pregnant women with T2DM, GDM and MGH

| SFH = 1.082 + 0.966*semana | | | |
| --- | --- | --- | --- |
| SFH (LL) = 0.629 + 0.95*week  SFH (UL) = 1.535 + 0.981*week | | | |
|  |  | 95%CI* | |
| Week | Expected SFH | LL | UL |
| 13 | 13.64 | 12.98 | 14.29 |
| 14 | 14.61 | 13.93 | 15.27 |
| 15 | 15.57 | 14.88 | 16.25 |
| 16 | 16.54 | 15.83 | 17.23 |
| 17 | 17.50 | 16.78 | 18.21 |
| 18 | 18.47 | 17.73 | 19.19 |
| 19 | 19.44 | 18.68 | 20.17 |
| 20 | 20.40 | 19.63 | 21.16 |
| 21 | 21.37 | 20.58 | 22.14 |
| 22 | 22.33 | 21.53 | 23.12 |
| 23 | 23.30 | 22.48 | 24.10 |
| 24 | 24.27 | 23.43 | 25.08 |
| 25 | 25.23 | 24.38 | 26.06 |
| 26 | 26.20 | 25.33 | 27.04 |
| 27 | 27.16 | 26.28 | 28.02 |
| 28 | 28.13 | 27.23 | 29.00 |
| 29 | 29.10 | 28.18 | 29.98 |
| 30 | 30.06 | 29.13 | 30.97 |
| 31 | 31.03 | 30.08 | 31.95 |
| 32 | 31.99 | 31.03 | 32.93 |
| 33 | 32.96 | 31.98 | 33.91 |
| 34 | 33.93 | 32.93 | 34.89 |
| 35 | 34.89 | 33.88 | 35.87 |
| 36 | 35.86 | 34.83 | 36.85 |
| 37 | 36.82 | 35.78 | 37.83 |
| 38 | 37.79 | 36.73 | 38.81 |
| 39 | 38.76 | 37.68 | 39.79 |
| 40 | 39.72 | 38.63 | 40.78 |
| 41 | 40.69 | 39.58 | 41.76 |
| 42 | 41.65 | 40.53 | 42.74 |
|  |  |  |  |

*LL = lower limit

UL = upper limit
