# Supplementary material for: Symphysis-Fundal Height Curve in Pregnancies Complicated by Maternal Hyperglycemia: Comparison with Curves of Nondiabetic Pregnant Women
Source: Biomed Res Int. 2020 Sep 1;2020:1908764. doi: 10.1155/2020/1908764 (PMC7481913; doi:10.1155/2020/1908764)
Supplement: Supplementary Materials — Table 1A: glycemic control assessed between 13 and 39 weeks among pregnant women with T2DM, GDM, and MGH. Table 2A: SFH expected value with upper and lower limits (95% CI) between 13 and 42 weeks or gestation among pregnant women with T2DM, GDM, and MGH. [file 1908764.f1.zip › mat.1908764.v2.pdf]

Biomed Research International

Symphysis-fundal height curve in pregnancies complicated by maternal hyperglycemia – comparison with curves of non-diabetic pregnant women (#1908764)

Supplementary materials

Table 1A. Glycemic control assessed between 13 and 39 weeks among pregnant women with T2DM, GDM and MGH

Weeks	# glucose tests	minimum	maximum	mean (mg/dL)
13	27	87.60	233.30	123.12
14	33	72.40	213.00	120.65
15	25	70.40	189.60	**118.60**
16	35	87.90	186.00	**117.11**
17	31	69.60	249.10	**110.71**
18	33	67.30	170.30	**109.62**
19	37	66.30	161.90	**112.84**
20	40	69.70	216.80	**116.52G**
21	42	73.20	240.00	**111.31**
22	46	80.00	170.80	**115.08**
23	46	72.20	273.50	123.94
24	69	76.20	174.00	**111.01**
25	76	76.30	180.80	**112.06**
26	88	79.70	188.50	**110.37**
27	83	75.10	334.70	**113.62**
28	116	56.40	235.50	**111.93**
29	89	79.20	157.30	**108.89**
30	110	54.00	281.80	**109.35**
31	109	72.00	198.00	**109.56**
32	139	61.80	198.20	**109.01**
33	145	76.80	236.30	**106.53**
34	149	52.80	152.50	**104.14**
35	167	67.40	180.90	**100.91**
36	163	58.80	187.50	**99.31**
37	124	64.10	138.00	**98.38**
38	41	77.50	124.00	**100.16**
39	11	81.10	106.30	**94.46**

Table 2A. SFH expected value with upper and lower limits (95% CI) between 13 and 42 weeks or gestation among pregnant women with T2DM, GDM and MGH

SFH = 1.082 + 0.966*semana

SFH (LL) = 0.629 + 0.95*week
SFH (UL) = 1.535 + 0.981*week

		95%CI*	
**Week**	Expected SFH	LL	UL
**13**	13.64	12.98	14.29
**14**	14.61	13.93	15.27
**15**	15.57	14.88	16.25
**16**	16.54	15.83	17.23
**17**	17.50	16.78	18.21
**18**	18.47	17.73	19.19
**19**	19.44	18.68	20.17
**20**	20.40	19.63	21.16
**21**	21.37	20.58	22.14
**22**	22.33	21.53	23.12
**23**	23.30	22.48	24.10
**24**	24.27	23.43	25.08
**25**	25.23	24.38	26.06
**26**	26.20	25.33	27.04
**27**	27.16	26.28	28.02
**28**	28.13	27.23	29.00
**29**	29.10	28.18	29.98
**30**	30.06	29.13	30.97
**31**	31.03	30.08	31.95
**32**	31.99	31.03	32.93
**33**	32.96	31.98	33.91
**34**	33.93	32.93	34.89
**35**	34.89	33.88	35.87
**36**	35.86	34.83	36.85
**37**	36.82	35.78	37.83
**38**	37.79	36.73	38.81
**39**	38.76	37.68	39.79
**40**	39.72	38.63	40.78
**41**	40.69	39.58	41.76
**42**	41.65	40.53	42.74

*LL = lower limit

UL = upper limit
